# Case report: Robotically visualized and biopsy-confirmed peritoneal carcinomatosis as initial identification of metastatic prostate adenocarcinoma in a patient with a history of prostatic urethral lift

**DOI:** 10.3389/fonc.2023.1284688

**Published:** 2024-01-19

**Authors:** Qateeb Khan, Bryn Myers, Breann Bowar, Maryam Khan, Henry Mullaney, Jordan Gainey, Robert Schneider, Laila Dahmoush, Kenneth G. Nepple, James D. Byrne

**Affiliations:** ^1^ Department of Radiation Oncology, University of Iowa Hospitals and Clinics, Iowa City, IA, United States; ^2^ Carver College of Medicine, Iowa City, IA, United States; ^3^ Marquette University, Milwaukee, WI, United States; ^4^ Basic Biomedical Sciences Program, University of South Dakota Sanford School of Medicine, Vermillion, SD, United States; ^5^ Department of Hospital Dentistry, University of Iowa Hospitals and Clinics, Iowa City, IA, United States; ^6^ Department of Pathology, University of Iowa Hospitals and Clinics, Iowa City, IA, United States; ^7^ Department of Urology, University of Iowa Hospitals and Clinics, Iowa City, IA, United States

**Keywords:** prostate cancer, RALP, robot-assisted laparoscopic radical prostatectomy, metastatic prostate cancer (mPCa), peritoneal metastases

## Abstract

**Background:**

Peritoneal carcinomatosis is a particularly rare presentation of prostate cancer. Here we report a rare clinical case of surgically identified peritoneal carcinomatosis at the time of a planned robotic prostatectomy in a patient with a history of prostatic urethral lift procedure.

**Case presentation:**

A 72-year-old man, with a history of urinary retention managed with tamsulosin, presented to his local urologist. Prostatic urethral lift procedures were performed for symptom management. After a definitive uptrend in his prostate-specific antigen (PSA) values, a biopsy was obtained, which demonstrated prostate adenocarcinoma. On presurgical multidisciplinary review, it was presumed that he had very high-risk localized prostate cancer. However, upon initiation of robotically assisted laparoscopic radical prostatectomy (RALP), he was noted to have numerous punctate white plaques on the peritoneum; biopsy of these lesions confirmed metastatic disease—for which the patient was starting on triple therapy per the PEACE-1 trial. The PSA level responded appropriately, decreasing from 16.8 to 0.08. Genetic testing was performed and returned negative for any clinically significant mutations.

**Conclusion:**

Our patient, diagnosed with peritoneal carcinomatosis during a planned RALP, highlights the importance of vigilant laparoscopic exam prior to this prostatectomy. Multidisciplinary discussion is crucial for individualized and optimal treatment planning.

## Introduction

Prostate cancer can have a wide variety of presentations, from localized to regional to metastatic disease. The majority of prostate cancers are diagnosed at a localized or regional stage. Per the Centers for Disease Control, only 5% of newly diagnosed prostate cancer cases are metastatic at presentation (1). However, the incidence of metastatic prostate cancer has increased in the past decade. Among these metastases, 84% are osseous in nature; other common sites of metastasis include the lymph nodes (10.6%), liver (10.2%), and chest (9.1%) (2). Peritoneal carcinomatosis is rarely clinically seen. In this report, we describe a case of biopsy-confirmed peritoneal carcinomatosis discovered at the time of a planned, aborted robotically assisted radical prostatectomy (RARP).

## Case description

A 72-year-old man, with a history of urinary retention managed with tamsulosin, presented to his local urologist. Evaluation noted prostate-specific antigen (PSA) values of 1.8 ng/mL, 7.3 ng/mL, 2.7 ng/mL, 2.4 ng/mL, 4.98 ng/mL, and then 0.83 ng/mL. Although elevated PSA values were initially noted, given the downtrend, symptomatic management of urinary retention and nocturnal enuresis was pursued. The digital rectal examination (DRE) was unremarkable with a 30–40-g firm prostate that was normal feeling with no nodules, indurations, or tenderness. Seminal vesicles were not swollen, and no masses were detected. The initial International Prostate Symptom Score (IPSS) was noted to be 11, consistent with moderate lower urinary tract symptoms. The post-void residual volume (PVR) was 537 mL. A microscopic urinalysis with culture was performed and returned negative. Given these findings, office cystoscopy was performed and showed moderate lateral prostate enlargement with mild intravesical protrusion and no evidence of bladder cancer.

Given his lower urinary tract symptoms, the patient had a prostatic urethral lift (UroLift or PUL) procedure performed with four implants. This was performed transurethrally, using a cystoscope with prostatic implants that lift and hold the enlarged prostate tissue to decrease obstruction. He was noted to have a marked decrease in his lower urinary tract symptoms with a repeat IPSS resulting at 5, consistent with mild symptoms. PSA values on yearly check afterward were 5.84 ng/mL, 6.9 ng/mL, and 10.9 ng/mL. Total and free PSA were ordered and returned as 6.9 ng/mL and 1.3 ng/mL, respectively, indicating a low risk of prostate cancer. The patient then underwent a right shoulder repair, after which he had increasing lower urinary tract symptoms in terms of frequency, weak stream, and incomplete emptying. He therefore underwent a repeat PUL procedure performed with six implants and was noted to have a significant decrease in the burden of symptoms. The IPSS was noted to be 2.

**Figure 1:**
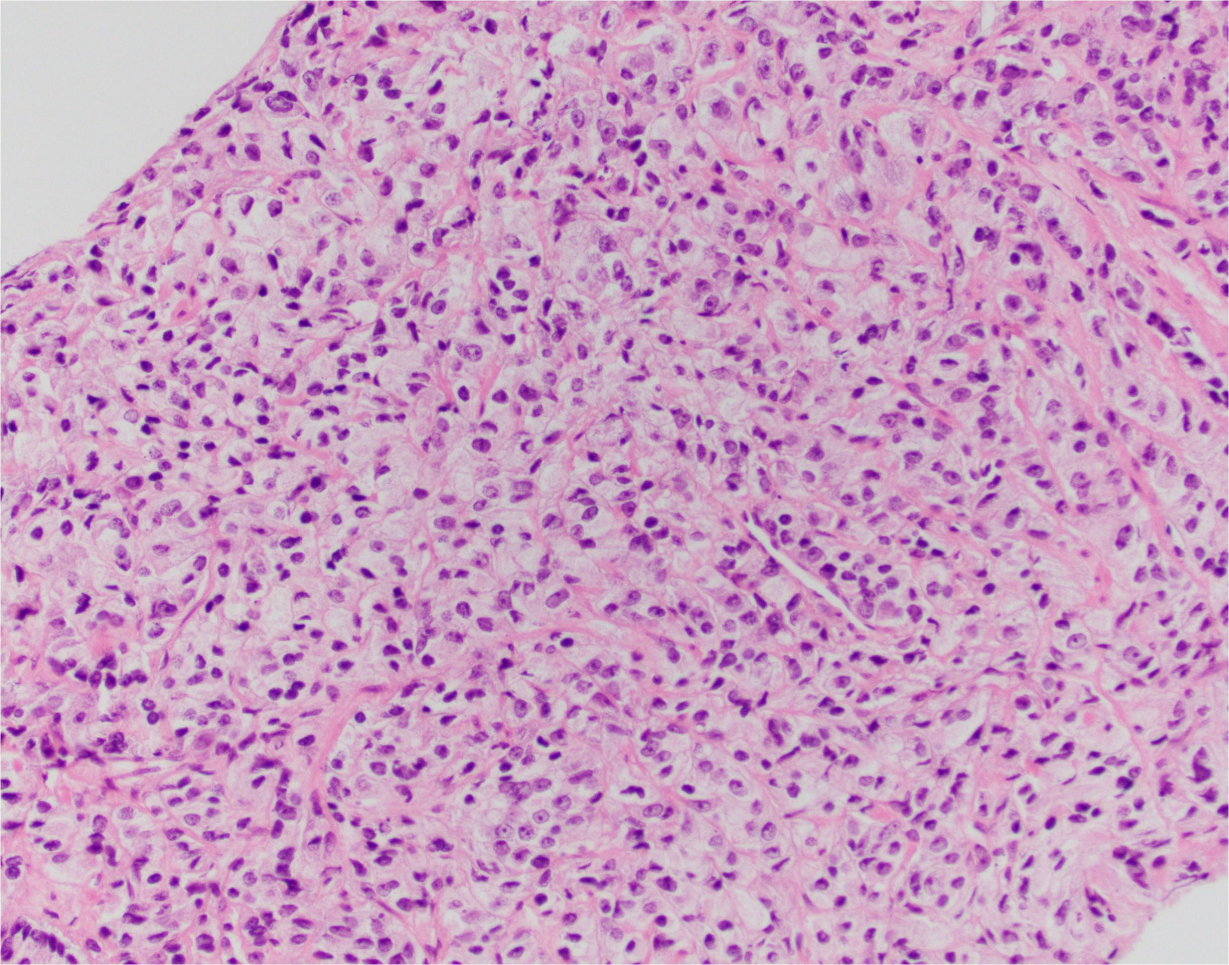
S-22-9401: prostate core showing adenocarcinoma, Gleason score 5 + 5 = 10 (grade group 5).

**Figure 2:**
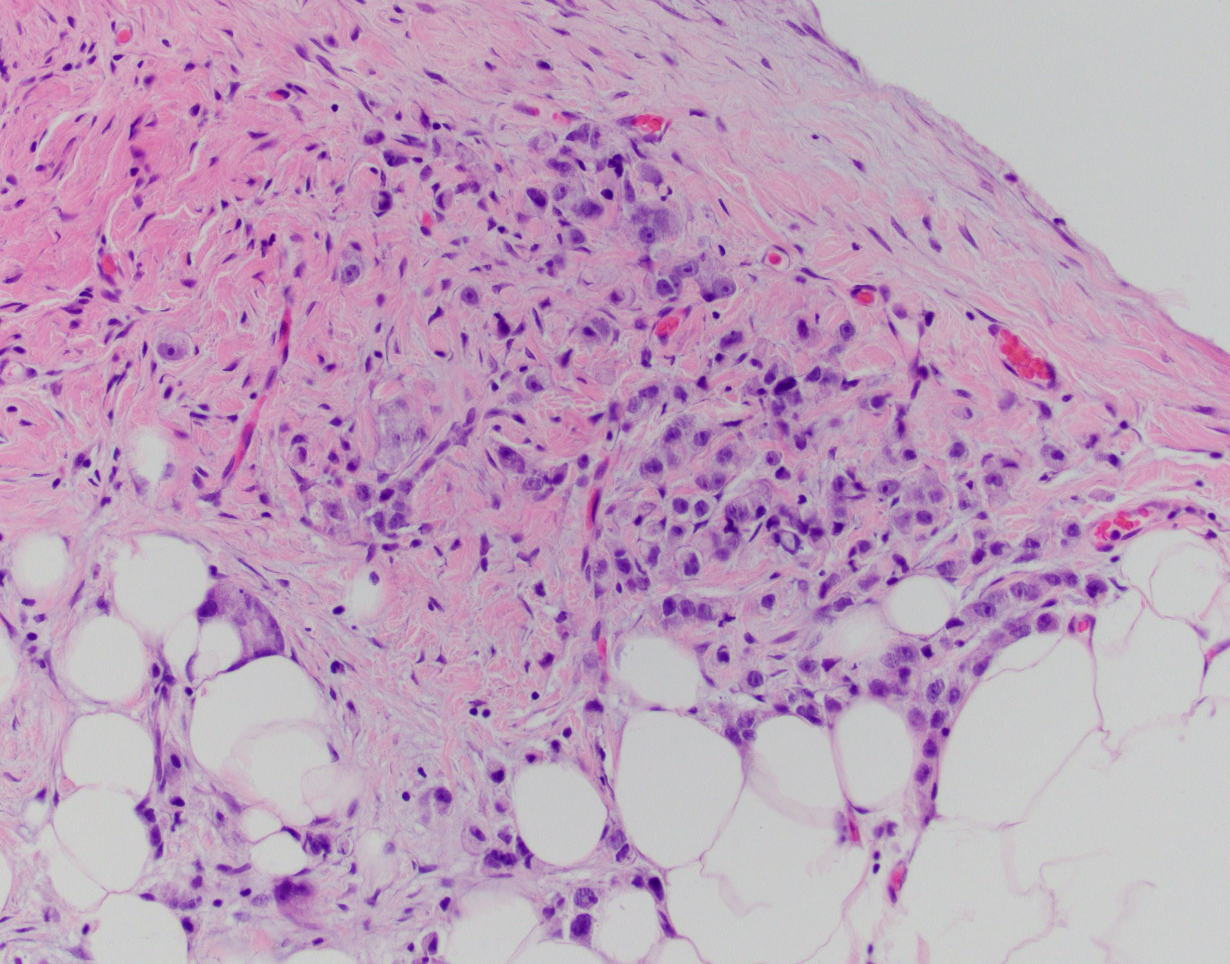
S-22-12486-2: peritoneal biopsy with metastatic adenocarcinoma, identical to the prostatic tumor.

**Figure 3:**
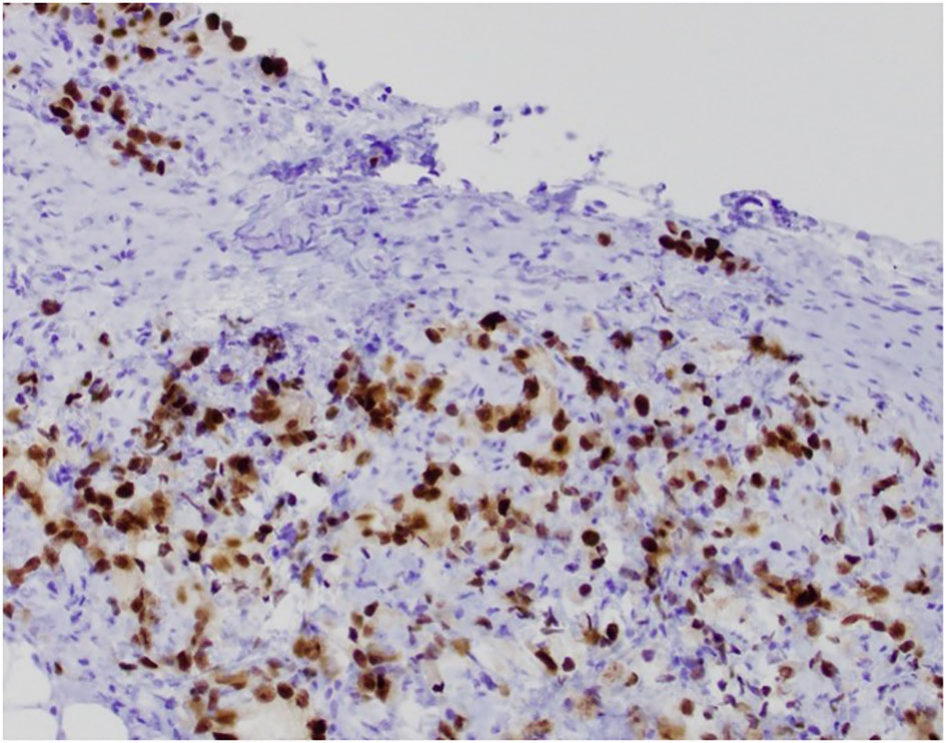
S-22-12486-3: peritoneal biopsy with tumor cells positive for NKX3.1 immunostain, confirming prostatic origin.

After 11 months, the PSA value was 12.89 ng/mL. At this point, given the trend in PSA values, a transrectal ultrasound-guided biopsy was performed. Pathology returned as Gleason 5 + 5 = 10 in 1/12 cores (70% of core), 5 + 4 = 9 in 9/12 cores (up to 100% of core), and 4 + 5 = 9 in 2/12 cores (up to 95% of core) (**Figures 1**–**3**). All 11 biopsy sites contained grade group 5 disease involving at least 70% of all cores. The PSA density at that time was 0.20 ng/mL/cc. He was then referred to the Holden Comprehensive Cancer Center at the University of Iowa.

Given the high-risk pathologic features, F-18 PyL prostate-specific membrane antigen positron emission tomography scan computed tomography (F-18 PyL PSMA PET/CT) was obtained and demonstrated abnormal focal uptake in the left prostate, as well as a small focus in the midline of the prostate. There was no abnormal uptake of F-18 PyL elsewhere to suggest metastatic disease. Radiology reported widespread osseous lesions without uptake, reported as likely representing osteopoikilosis: “spotted bone” as a result of an inherited autosomal dominant benign osteosclerotic dysplasia of the bones. A subsequent bone scan reported suspected metastatic prostate cancer involving the pelvis, hips, and the midshaft of left femur. However, a CT-guided bone biopsy of the right iliac lesion was performed and was negative for malignancy.

An MRI of the prostate was performed, which showed diffuse T2 hypointensity of the entire prostate and bilateral medial seminal vesicles. Suboptimal diffusion-weighted images and metal artifact precluded the assessment of Prostate Imaging Reporting and Data System (PI-RADS) scoring. Focal extracapsular extension (ECE) was noted in the right posterior lateral mid-peripheral zone, suspicious for neurovascular bundle involvement. There was no lymphadenopathy.

**Figure 4:**
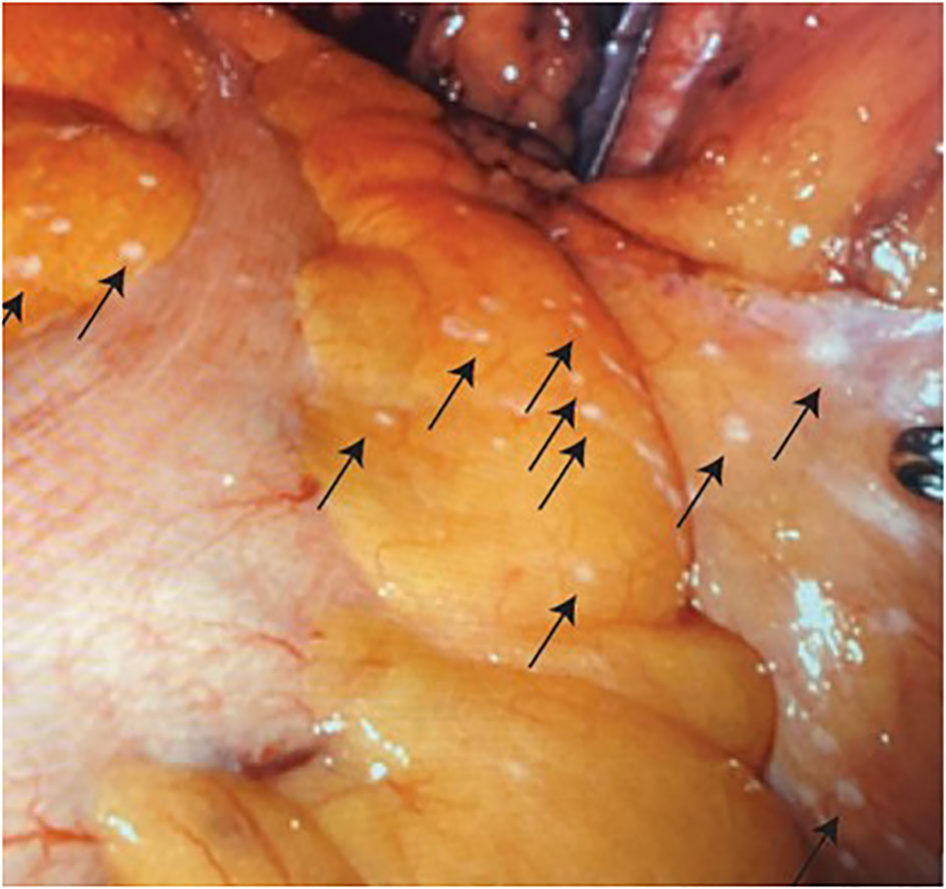
Numerous punctate white tumor deposits (indicated by arrows) on the peritoneum and epiploicae of the sigmoid colon.

Given the negative bone biopsy findings in conjunction with lack of activity in bone lesions on PyL PSMA PET/CT, the patient was presumed to have localized very high-risk prostate cancer per National Comprehensive Cancer Network (NCCN) risk stratification, version 4.2023; however, the possibility of metastatic disease was discussed. After shared decision-making, RARP with pelvic lymph node dissection was planned. Abdominal insufflation was established in the standard fashion using a Veress needle. Upon robotic visual evaluation, the patient was found to have numerous punctate white plaques on the peritoneum and epiploicae of the sigmoid colon (**Figure 4**). These findings were unusual for prostatectomy and were concerning for peritoneal carcinomatosis. They were biopsied by removing an area of peritoneum, which was sent for a frozen section procedure. The frozen section was reported as carcinoma. Given these findings and the absence of known benefit of prostatectomy in the setting of metastatic disease, radical prostatectomy was aborted, but a bilateral standard pelvic lymph node dissection was performed for diagnostic staging purposes with the borders as follows: external iliac vein superiorly, bladder medially, node of Cloquet distally, and obturator nerve inferiorly. There were no grossly enlarged pelvic lymph nodes identified during the dissection. Two left pelvic lymph nodes and five right pelvic lymph nodes were excised. Final pathology confirmed metastatic prostatic adenocarcinoma in an additional peritoneal sample. Lymph nodes were negative for malignancy; they demonstrated histiocytic inflammation consistent with the patient’s history of prior joint replacements. He was discharged on postoperative day 1.

The patient was discussed at the multidisciplinary tumor board. A subsequent review identified prior CT imaging from years prior without any bone lesions, which ruled out congenital osteopoikilosis and was thus consistent with widespread bone metastatic disease in addition to peritoneal carcinomatosis.

Therefore, for this patient, now diagnosed with high-volume metastatic disease, systemic therapy with androgen deprivation therapy was initiated. Leuprolide, five cycles of docetaxel (with the last cycle held due to hyperglycemia-related acidosis), and abiraterone was initiated per the PEACE-1 trial; no radiotherapy was initiated due to widespread disease and his prior history of significant lower urinary tract symptoms. The pretreatment PSA was 16.20. The patient responded to therapy with PSA values of 1.68 at 1 month, 0.14 at 6 months, and 0.08 at 1 year. He had genetic counseling performed, due to his metastatic prostate cancer; no clinically significant mutations were found using the 52-gene panel from Ambry Genetics Laboratory.

**Figure 5:**
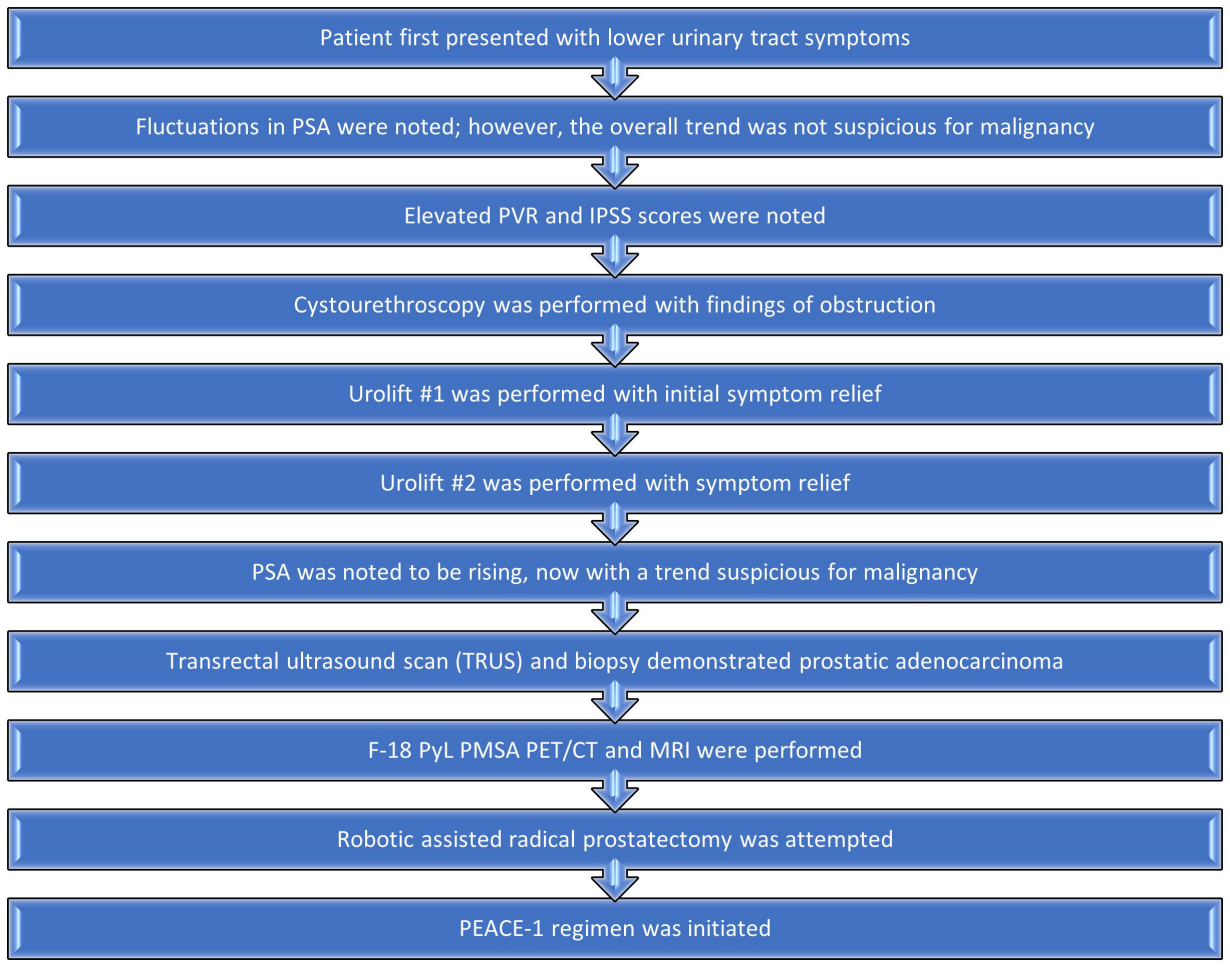
Diagnostic and therapeutic course.

A summary of the patient’s treatment course is outlined in **Figure 5**. From his perspective, the initial diagnostic tests performed while having fluctuations in his PSA caused some worry, dissatisfaction, and concern. However, after diagnosis, he was extremely content with his cancer treatment team. He is overall pleased with the marked downtrend in PSA with minimal side effects, i.e., mild fatigue, secondary to his treatment regimen.

## Discussion

The majority of prostate cancers present as locoregional disease. Patients that initially present with metastatic disease typically have osseous and/or nodal disease (2). In an analysis of 316 patients with metastatic spread, metastases in the abdominal cavity were seen in less than 1% of patients (3).

Development of peritoneal metastases after prostatectomy has been previously reported (4–8). Isolated peritoneal metastases without prior surgery or other definitive sites of metastatic disease are a more unusual phenomenon with only seven other case reports published on our literature review (8–14). An autopsy review article of 523 prostate cancer patients showed that 13 cases had peritoneal deposits without other metastatic sites (15).

Management of metastatic prostate cancer has been evolving. For many years, androgen deprivation therapy (ADT) alone was the standard of care for patients with *de novo* metastatic castration-sensitive prostate cancer. However, docetaxel was shown to improve survival when added to ADT (16). Similarly, abiraterone was also demonstrated to improve survival when added to ADT (17). The overall survival benefit of triplet therapy involving ADT, docetaxel, and abiraterone was demonstrated in the recent PEACE-1 trial (18). Therefore, per multidisciplinary discussion, given the diagnosis of *de novo* metastatic castration-sensitive prostate cancer, triplet treatment was initiated.

Of note in this presentation is the relatively low PSA in newly diagnosed metastatic castration-sensitive prostate cancer and the absence of PET avid disease on PyL PET/CT. Despite PSA testing being used for detection and surveillance of prostate cancer, PSA is an imperfect marker. This was particularly seen in this case with the wide variations in PSA throughout the patient’s clinical history presenting the clinicians with true diagnostic challenges in terms of benign prostate enlargement versus evolution of a malignant process. A Surveillance, Epidemiology, and End Results (SEER) analysis demonstrated that 5.6% of Gleason 8–10 tumors were diagnosed with PSA ≤ 2.5 ng/mL (19). On initial presentation, our patient’s PSA was consistently <10, until a PSA of 12.89 ng/mL prompted a prostate biopsy, and the PSA was not markedly elevated despite the Gleason 5 + 5 pathology. It has been reported that metastatic prostate cancer with serum PSA levels less than 10 ng/mL are associated with poorly differentiated or undifferentiated tumors and that these cancers have a worse prognosis compared with typical metastatic prostate cancer with lymph node, visceral, or bone metastases (20, 21). The low PSA could be due to the dedifferentiated biology of aggressive prostate cancer and lack of ability to produce PSA. Furthermore, elevated serum PSA is indicative of androgen receptor signaling-driven cell growth. In the event of low PSA with high-risk disease, the disease may not be mediated via this growth mechanism. It is known that hormone-sensitive prostate cancer can acquire androgen receptor mutations, including variants that can lead to castration-resistant prostate cancer (22). Furthermore, in our case, the peritoneal tumor deposits and bone disease were not detected by the PSMA PET/CT scan. This likely is due to the peritoneal lesions being too small to be detected and the fact that some bone lesions may not show uptake. However, only the disease on the left side of the prostate was avid, while bilateral biopsies showed grade group 5 disease. This could represent his disease as having a clonal expansion of non-PSA-emitting and non-PSMA avid disease. In the aforementioned SEER analysis, patients with Gleason 8–10 disease with PSA ≤ 2.5 ng/mL had a higher risk of prostate cancer–specific mortality than standard NCCN high-risk disease [adjusted hazard ratio (AHR) 1.92, 95% CI 1.18–3.14, P = 0.009; 47-month prostate cancer–specific mortality (PCSM) 14.0% vs. 10.5%]. They also tended to have an increased risk for all-cause mortality when treated with ADT and definitive radiotherapy (AHR 1.27, 95% CI 0.89–1.81, P = 0.194) compared to patients with PSA > 2.5 ng/mL (AHR 0.87, 95% CI 0.81–0.94, P < 0.001).

Also, of interest for this patient was the PUL procedures performed. PUL is a minimally invasive procedure performed through the urethra that consists of implants being placed through the prostate tissue to open the urethra in the setting of prostatic hypertrophy. The PUL implant consists of an outer prostatic capsular attached to a PET monofilament suture and then a urethral end piece. The UroLift device package insert includes documentation of adverse events including PSA elevations but no specific mention of prostate cancer risk. There are several reports in the literature of peritoneal carcinomatosis presenting after RARP, suspected to be the result of direct seeding of tumor cells in the peritoneal space during prostate removal (4, 6, 7). In line with this mechanism of cancer spread, there remains the question of whether the PUL procedure performed in our patient could have possibly contributed to the development of peritoneal metastases given the manipulation and disruption of the prostate architecture. Anatomically, the prostate resides in the extraperitoneal space, and direct spread from prostate capsular spread would not typically be expected to be intraperitoneal. This case does highlight that prostate cancer evaluation should be considered prior to PUL. Per our literature search, there is no report of peritoneal carcinomatosis following a PUL procedure. One systemic review and meta-analysis reported studies typically excluded patients with PSA > 10; however, some studies included this patient population if a negative prostate biopsy was obtained. Overall, the prostatic urethral lift outcome reports are focused on urologic symptoms and side effects but have not reported on prostate cancer adverse events (23).

In summary, we present a rare clinical case of surgically identified peritoneal carcinomatosis at the time of a planned robotic prostatectomy in a patient with a history of prostatic urethral lift procedure. The peritoneal carcinomatosis was found at the time of robotic scope insertion for a planned RALP, demonstrating the importance of vigilant laparoscopic exam prior to pelvic surgical procedures. Furthermore, we provided images of the small peritoneal implants as an example for surgical planning and learning.

## Data availability statement

The original contributions presented in the study are included in the article/supplementary material. Further inquiries can be directed to the corresponding author.

## Ethics statement

Written informed consent was obtained from the individual(s) for the publication of any potentially identifiable images or data included in this article.

## Author contributions

QK: Conceptualization, Writing – original draft, Writing – review & editing. BM: Conceptualization, Writing – original draft, Writing – review & editing. BB: Conceptualization, Writing – original draft, Writing – review & editing. MK: Writing – review & editing. HM: Writing – review & editing. JG: Writing – review & editing. RS: Writing – review & editing. LD: Writing – review & editing. KGN: Writing – review & editing. JDB: Writing – review & editing.
